# Recycled Household Ash in Rice Paddies of Bangladesh for Sustainable Production of Rice Without Altering Grain Arsenic and Cadmium

**DOI:** 10.1007/s12403-023-00539-y

**Published:** 2023-02-09

**Authors:** Yogesh Gupta, Mahmud Hossain, M. Rafiqul Islam, Md. Moyeed Hasan Talukder, Md. Atiqur Rahman Khokon, Mohammad Mahir Uddin, Humayun Kabir, Manus Carey, Kathryn Ralphs, Natalia Valadares de Moraes, Andrew A. Meharg, Caroline Meharg

**Affiliations:** 1 Institute for Global Food Security, School of Biological Sciences, Queen’s University Belfast, 19 Chlorine Gardens, Belfast, BT9 5DL UK; 2 School of Medicine, Dentistry and Biomedical Sciences, Queen’s University Belfast, 97 Lisburn Road, Belfast, BT9 7BL UK; 3 Department of Soil Science, Bangladesh Agricultural University, Mymensingh, Bangladesh; 4 Department of Plant Pathology, Bangladesh Agricultural University, Mymensingh, Bangladesh; 5 Department of Entomology, Bangladesh Agricultural University, Mymensingh, Bangladesh; 6 Department of Agricultural Economics, Bangladesh Agricultural University, Mymensingh, Bangladesh; 7 Department of Natural Products and Toxicology, School Pharmaceutical Sciences, Sao Paulo State University (UNESP), Rodovia Araraquara-Jau, Km 01, Araraquara, SP 14800-903 Brazil

**Keywords:** Ash, Arsenic, Cadmium, Rice, Paddy soil, Microbes

## Abstract

In Bangladesh most agronomic biomass (straw, husk, dried dung) is burnt for domestic cooking use. Consequently, the soil is continuously stripped of mineral nutrients and carbon (C) substrate. Here we investigate if recycling of household ash (ash) as fertilizer can sustainably improve soil fertility as well as minimise accumulation of toxic elements (As, Cd) in rice grain. Large scale field trials across two geographic regions (Barind, Madhupur) and two seasons (wet, dry) and with application of 3 fertiliser treatments (NPKS, ash, NPKS + ash) were conducted. At the end of each season, the impact of region*season*treatment on soil microbial comunities, rice yield, and grain quality (As, Cd, nutrient elements) was assessed. When compared to conventional field application rates of NPKS (control), application of ash boosted rice yield by circa. 20% in both regions during wet and dry season, with no effect on rice grain carcinogenic inorganic arsenic (iAs), dimethylarsonic acid (DMA) or cadmium (Cd), but with potential to increase zinc (Zn). For soil microbial communities, a significant region and season effect as well as correlation with elements in rice grain was observed, amongst these Cd, Zn, iAs and DMA. This study illustrates that application of ash can reduce the requirement for expensive chemical fertiliser, whilst at the same time increasing rice yield and maintaining grain quality, making farming in Bangladesh more sustainable and productive. The study also implies that the combined impact of region, season, and soil microbes determines accumulation of elements in rice grain.

**Supplementary Information:**

The online version contains supplementary material available at 10.1007/s12403-023-00539-y.

## Introduction

Rice is a staple food for more than half of the world’s population. In Bangladesh rice is cultivated throughout the year with the intensity of cultivation increasing year-by-year to meet the demands of an ever increasing population. The expansion of rice production has been achieved though the extensive use of chemical fertilizers (Islam et al. 2008; Tewatia et al. 2008; Sha et al. 2008). However, yield response to chemical fertilizer addition has been steadily reducing (Hossain et al. 2016; Islam et al. 2008; Tewatia et al. 2008; Sha et al. 2008). In Bangladesh, most of the agronomic (straw, stalk, husk and manure) residue is burned for household fuel, leading to decreased soil fertility through multi-element deficiencies and lack of organic matter content (Hossain et al. 2016; Islam et al. 2008; Tewatia et al. 2008; Sha et al. 2008). Hence, yield reduction is due to the enhanced cropping intensity combined with the failure to recycle crop residues. New approaches are needed to enhance crop yields to feed Bangladesh’s populace, but any intervention must be of low cost and sustainably use local resources to be achievable (Hossain et al. 2016; Islam et al. 2008; Tewatia et al. 2008; Sha et al. 2008).

Household ash (ash) is available in Bangladesh with sufficient amount to be repositioned as a fertilizer, being rich in P at percent concentrations, as well as Ca, K, Mg and Si and wide-range of micro-nutrients (Braadbaart et al. 2012; Thy et al. 2013). Recycling of ash as fertilizer has potential to sustainably improve soil fertility (Braadbaart et al. 2012; Thy et al. 2013). While there is little tradition of ash fertilizer use in Bangladesh (Sha et al. 2008), elsewhere, it was widely employed (Meharg and Meharg 2015). Straw and straw ash return to soil has been shown to increase yield of crops (Tewatia et al. 2008; Thind et al. 2012; Okon et al. 2005; Saleque et al. 2002), promote soil microbial activity (Jin et al. 2020), and is seen as a potential route towards a more sustainable production of rice grain (Jin et al. 2020; Tewatia et al. 2008; Thind et al. 2012; Okon et al. 2005; Saleque et al. 2002).

Rice efficiently assimilates arsenic (As), a class 1 non-threshold carcinogen, and cadmium (Cd), a nephrotoxin and carcinogen into grain, and is the main source globally of these elements to the human diet (Meharg et al. 2009; Shi et al. 2020), as elevated arsenic in drinking water, the other major contributor, only occurs in a limited number of regions (Meharg and Zhao 2012). Elemental concentration and redox potential affect their uptake into rice, with the latter having opposite effect on As and Cd bioavailability (Carey et al. 2020; Chowdhury et al. 2017; Zhao et al. 2020). During the dry season (Boro), irrigation results in constant flooding. This favours anaerobic soil microbial communities responsible for As reduction and methylation, leading to As mobilization and increased uptake by root that result in elevated As concentrations in rice grain (Chowdhury et al. 2017; Zhao et al. 2020). During the wet season (Amon), irrigation is achieved via rainfall resulting in less constant flooding, leading to Cd uptake being more prevalent as its biovailability is greater under more aerobic soil conditions (Chowdhury et al. 2017; Zhao et al. 2020). Similarly, alternate flooding and drying was shown to decrease As but increase Cd in rice grain (Norton et al. 2017). Other soil elements also need to be considered, in particular Fe, as As is sequestered in iron plaque around rice roots (Hu et al. 2015). In this context impact of soil microbial communities should also be investigated as they drive these chemical processes (Chauhan et al. 2018; Duan et al. 2020; Hu et al. 2015; Hussain et al. 2021; Lami et al. 2013; Luo et al. 2019; Norton et al. 2017; Sebastian and Prasad 2014; Zeng et al. 2020; Zhao et al. 2020).

Here we report on field experiments with 3 different P fertilizer treatments (NPKS, ash, NPKS plus ash) over 2 growing seasons (dry and wet) at field sites in two geographically distinct regions of the Bangladesh (Madhupur and Barind tract). The objectives were (1) to identify the effect of ash addition on rhizosphere microbial communities and rice yield; (2) to determine regional (Madhupur and Barind tract) and seasonal (wet and dry season) differences in rhizosphere microbial communities and rice yield; and (3) to establish a potential link between accumulation of elements in rice grain (As and Cd, as well as nutrients) with region, season, treatment and/or rhizosphere microbial communities.

## Material and Methods

### Study Area

The study was conducted across an area of 12,000 km^2^ on the Pleistocene Tracts of Bangladesh, Barind and Madhupur. This area encompass 7% of the country’s total land area, and is subject to extensive agricultural activity due to their soils geomorphological settings (Brammer 1996). The soil classification for both study regions are Fluvisols. These physiographical units were selected as their soils are naturally more nutrient deficient and lower in pH compared to adjacent Holocene floodplain soils (Moslehuddin et al. 1997). This is because Pleistocene soils do not receive fresh yearly inputs of nutrient rich fluvial deposits and due to age related weathering. Eighteen subsistence farming communities were studied. Of these, 9 villages were located in the western Barind Tracts and 9 in the eastern Madhupur Tracts, with co-ordinates for the location of each field trial given in Table 1.

**Table 1:** Ash and fertilizer application rates for the Madhupur and Barind Tracts

		Latitude	Longitude	Urea (kg/ha)	TSP (kg/ha)	MoP (kg/ha)	gypsum (kg/ha)	ash (kg/ha)
**Madhupur Tract**								
NPKS				226	150	176	113	0
NPKS plus ash				226	150	176	113	1000
ash								
Farmers’ Field	Bandiya	24.4405	90.3562	300	0	156	88	1140
	Madwary	24.4424	90.3690	276	0	0	50	1230
	Voradova	24.4464	90.3807	216	0	48	28	984
	Menjana	24.4472	90.3167	288	0	64	76	1316
	Bonkuya	24.4475	90.3187	276	0	12	44	1340
	Bogajan	24.4513	90.3306	300	0	20	16	1780
	Kholabari	24.4586	90.2857	300	0	0	20	2100
	Borayed	24.4618	90.3300	325	0	0	0	3020
	Borojoyna	24.4770	90.2955	336	0	40	52	2183
**Barind Tract**								
NPKS				216	60	60	30	0
NPKS plus ash				216	60	60	30	1000
ash								
Farmers’ Field	Jevoal	24.5677	88.5790	300	0	148	84	2000
	Chanpur	24.5704	88.5691	300	0	140	40	2000
	Mothurapur	24.5712	88.5761	288	0	100	60	488
	Gollahpara	24.5850	88.5699	264	0	0	28	2000
	Gokul	24.6158	88.5866	274	0	0	50	3281
	Mothura	24.6248	88.5848	288	0	0	16	3200
	Thalanda	24.6292	88.5843	288	0	96	52	2000
	Sumaspur	24.6361	88.5836	300	0	184	68	2000
	Horidepur	24.6391	88.5691	300	0	128	36	2000

Both triple superphosphate (TSP) muriate of phosphate (MoP) was used as the P-fertilizer

### Field Trials

The field study ran for 1.5 years. In each region ash was collected from April 2017 and during the first experiment. The first batch of ash was applied to the dry season (Boro, groundwater irrigated) rice cultivation experiment that started in winter 2017 and the second batch to the wet season (Amon, rain-fed) rice cultivation experiment that started in August 2018. For 9 households in each of the 18 villages used in the study, the nature and frequency of biological materials used in domestic cooking was assessed. On the day of survey, the local field-team determined the weight of each biomass type burnt per household and all ash produced that day by each household was collected. The survey also quantified if ash was currently used agronomically. In addition, the plot size for each household farm was ascertained. The ash from each household was thoroughly mixed and sub-sampled for subsequent analysis. The remaining ash was bulked for all households per village, homogenized and stored in plastic bags for use in field trials. There was little difference in ash composition between season for each region.

For field trials in both regions, the 2 most commonly used, high-yielding rice cultivars were used, BRRI dhan28 for the dry, and BRRI dhan49 for the wet season. Experiments were conducted on 5 m*5 m subplots of 1 field, with 3 treatments * 3 replicates per treatment. Dry and wet season trials were conducted using the same fertilization plots, *i.e.* the same treatments were applied to each plot for both seasons. The treatments were: (1 = NPKS) conventional field application rates of NPKS, (2 = ash) ash to supply all required P plus additional supplementations of NPKS to make up to recommended rate, and (3 = NPKS + ash) conventional field application rates of NPKS with additional of 1 t/ha ash. Triple superphosphate (TSP), and muriate of potash (MoP), gypsum and urea where used as the source of NPKS fertilizer. To minimize fertilizer use, we integrated a “soils-based fertilizer” test developed in Bangladesh where simple test for available NPKS are used to modify, usually downward, fertilizer application (FRG 2012). FRG (2012) protocol was only applied to the ash treatment 2, as treatments 1 and 3 used farmers traditional urea application rate. Thus, the ash treatment 2 actually had more urea added compared to as the farmers practice due to use of FRG protocols for this treatment (Table 1). In effect, farmers are under N-fertilizing according to the FRG protocols and this must be born in mind in interpreting the data. For treatment 3, 1 t/ha was used as an application rate as this was achievable through ash produced per household (additional file 1 Table S3) plus wider community contribution (i.e. from rice mills, tea vendors, large farm owners). In Barind track median ash produced per year was 0.83 t/ha, though lower on the Madhupur tract at 0.56 t/ha. Ash was mixed by shovel in the field for application.

During experiment 1 (dry season) and 2 (wet season), rhizosphere soil closely adhering (released by the excabated roots after vigorous handshaking of stems for 30 s) to the root of plants was collected during the grain filling stage, transferred into 2 ml Eppendorf tubes on dry ice and subsequently stored in a − 80 ℃ freezer for DNA extraction. Rice straw and grain yields were recorded at harvest, dried and stored for subsequent chemical analysis, with grains dehusked before drying. At the end of the overall experiment, soil was sampled from each plot at harvest to a depth of 0–20 cm, using a 5 cm diameter corer, from 4 locations on the diagonal of the plot, 2.5 m from the centre. Subsequently the soil was bulked to provide a single sample per plot. On return to the laboratory soil was air dried for storage until further processing for analysis.

### Analytical Chemistry Methodologies

Characterisation of wholegrain for elements (Cd, Cu, Mn, P, Rb, Zn) and wholegrain As speciation (DMA, iAs) was conducted with ICP-MS and IC-ICP-MS, respectively, as previously described (Rahman et al 2019). Characterization of soils for elements (Al, As, Ba, Ca, Cl, Cu, Fe, K, Mg, Mn, Ni, P, Rb, S, Si, Sr, Zn) was conducted by XRF (Nex CG, Rigaku, Japan) as previously described (Sun et al. 2019). Soil pH was determined on a 2:1 distilled-deionized water:soil slurry. Characterization of ash for elements (Al, As, Ba, Ca, Cu, Fe, K, Mg, Mn, Ni, P, Rb, S, Si, Sr, Ti, Zn) was conducted by XRF (Nex CG, Rigaku, Japan) as previously described (Sun et al. 2019). Note that ash will be homogenised in the field and not 0.5 mm sieved, and larger organic debris, not quantified, will also be present. Therefore, the XRF analysis is on an operationally defined sub-sample of the ash and this must be considered when interpreting the data. For soil elemental analysis, air dry soil was 2 mm, and ash 0.5 mm, sieved. All material for chemical analysis was freeze dried and then powderised in a ZrO_2_ lined vessel and planetary ball milling. Appropriate soil CRMs (ISE921, NCS 73007), and rice (NIST 1568b), were used throughout and recoveries reported (additional file 1: Table S1).

### DNA Extraction and 16S rRNA Amplicon Sequencing Data Analysis

DNA was extracted from 0.5 g soil of 108 samples (2 regions * 9 villages * 2 seasons * 3 treatments), plus 1 negative control, using DNeasy PowerLyser PowerSoil kit DNA Isolation Kit (Qiagen) and quality checked via spectrophotometry (Nanodrop ND1000; Thermo Scientific, USA) and gel electrophoresis. The 250 bp paired end 16S amplicon sequencing on the Illumina Miseq was performed according to Coparaso et al. (2011).

The amplicon sequencing data was processed with the Qiime2-2019.04 pipeline (Bolyen et al. 2019). DADA2 (Callahan et al. 2016) was used for denoising and assembly into amplicon sequence varients (ASVs) and representative ASVs annotated according to the SILVA reference database (Quast et al. 2012) using the q2-feature-classifier with consensus-blast option. Subsequently the Qiime2 ASVs count table, taxonomy and metadata files were exported into tab-delimilated format for statistical analysis and plotting in R v4.1.1 (www.R-project.org) with R package phyloseq 1.36.0 (McMurdie and Holmes 2013), DESeq2 1.32.0 (Love et al. 2014), vegan 2.5.7 (Oksanan et al. 2018), pheatmap version 1.0.12 (https://cran.r-project.org/web/packages/pheatmap), corrplot version 0.84 (Wei and Simko 2021) and Prism 8.4 (Graphpad Software, San Diego, California, USA, www.graphpad.com) as described below.

Effect of region, season and treatment on microbial community composition was assessed by permutational analysis of variance on the Bray–Curtis distances with vegan, function adonis (Anderson 2008). Relative abundance (RA) data was generated with phyloseq, function tax_glom and transform_sample_counts, and plotted with Prism 8.4. Significant differences (region, season) of abundant (> 1%) phylum level ASVs were investigated with a t-test using the False Discovery Rate (FDR) < 0.05 as cutoff for significance (Prism 8.4). Significant differences for genera level ASVs (region, season) were identified with DESeq2 as described for microbiome applications (McMurdie and Holmes 2014) using the FDR < 0.01, absolute log2FC > 0.5 and basemean > 10 as cutoff for significance and a heatmap for selected results generated with pheatmap.

### Statistical Analysis and Integration of Chemical And Microbial Data

For analysis of the ash and grain yield data, parametric (normal distribution) and non-parametric (non-normal distribution) statistics (Mintab v. 19.2.0.0 (USA)) were used as appropriate. Statistical analysis of the soil elemental (two-way ANOVA) and grain elemental data (three-way ANOVA) and posthoc Tukey test were performed with R function lm (Chambers 1992), aov (Chambers et al. 1992), HSD.test, TukeyHSD (Miller 1981) and t.test. Boxplots were generated with Prism 8.4. Canonical analysis of principle coordinates (CAP) (Anderson and Willis 2003) was performed with R function ordinate (cap method) and plot_ordination (McMurdie an Holmes 2013) and Spearman’s correlation analysis between genus level ASVs and grain elemental data (P, Mn, Cu, Zn, Rb, Cd, DMA, iAs) with R function rcorr. Selected significant (*P* > 0.05) correlations were plotted with R function corrplot (Wei and Simko 2021).

## Results

### Ash and Soils

**Fig. 1:**
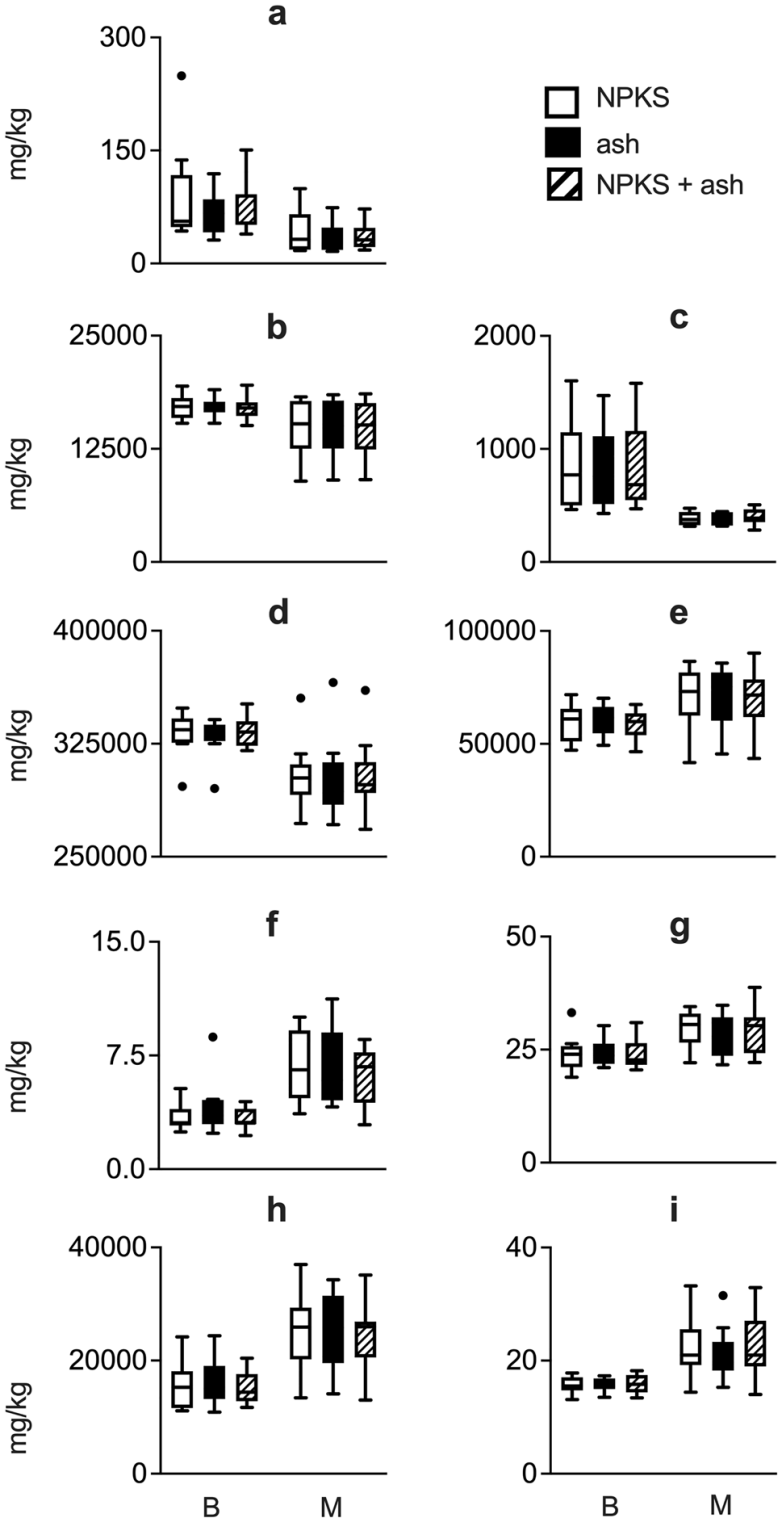
Boxplot for soil elemental analysis (XRF). Boxplot with whiskers (Tukey) for results obtained for soil elemental analysis (XRF) that was conducted on soil sampled at the end of the overall experiment from all treatment plots in the Madhupur and Barind region for elements **a** Cl, **b** K, **c** P, **d** Si, **e** Al, **f** As, **g** Cu, **h** Fe, **i** Ni. All units are in mg/kg. *B*  Barind region, *M*  Madhupur region, *NPKS*  traditional NPKS treatment, *ash*   ash treatment, *NPKS + ash*  traditional NPKS treatment plus ash. For details on significant statistical differences on soil chemical data please see additional file 1 table S4

The survey found that the farmers on the Barind tract produced more ash (0.58 t/y) than those from Madhupur (0.26 t/y), additional file 1: Fig. S2. The median farm size in the Barind region was 0.54 ha and in the Madhupur region 0.4 ha. These produced 0.83 t/ha ash and 0.56 t/ha ash (*P* < 0.01), respectively. Both regions used mixed and diverse fuel sources for ash production, with a preference for cow dung in the Barind (*P* < 0.001) versus scavenged branches (*P* < 0.001) and leaves (*P* < 0.001) in the Madhupur region. More fuel was burnt in the Barind Tract (*P* < 0.001), and this may reflect differences in fame income, being ~ 30% higher in the Barind region (*P* < 0.001), with a similar difference in fam size (*P* < 0.001). Elemental composition of ash showed strong regional differences for all elements investigated (except for P and Ti), with seasonal differences only observed for Mg, Ni and Si and region*season effect for P (additional file 1: Table S3). Soil chemical properties measured at the end of the overall experiment showed no effect of ash treatment but a highly significant difference between regions (additional file 1: Table S4). Soil pH (*P* < 0.01) and Cl, K, P, Si (*P* < 0.001) were significantly higher in the Barind (Fig. 1a–d), whereas Sr, Ca (*P* < 0.05) Al, As, Cu, Fe, and Ni (*P* ≤ 0.001) were significantly higher in the Madhupur region (Fig. 1e–i).

### Rice

**Fig. 2:**
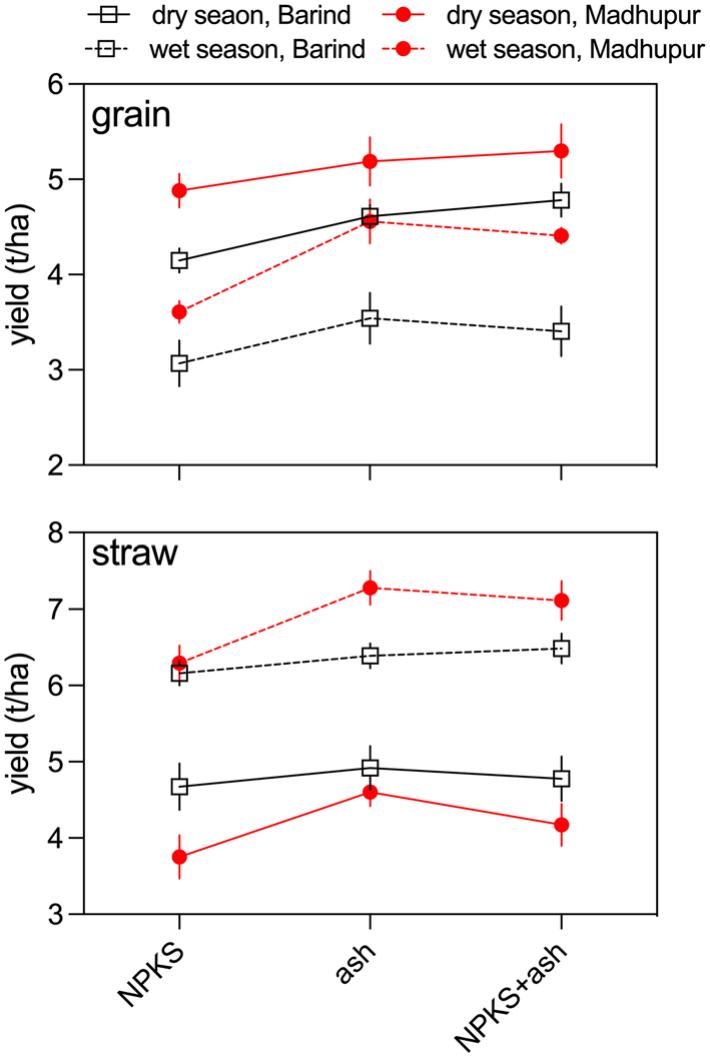
Grain and straw yields in response to treatments. The graphs show the grain and straw yields obtained for for each experimental treatment, across two regions and both seasons. Symbols represent the average of the nine sites at each location, and bars are the standard error of the mean

When compared to conventional NPKS (control), grain yield responded positively to ash and NPKS + ash treatment throughout both seasons in both regions (Fig. 2). Grain yield typically increased by 0.5 t/ha, an improvement of 20% and showed highly significant region, treatment and season*treatment effect (*P* < 0.001). The largest percentage increases in grain yield were found for wet season on the Madhupur Tract were a 26% increase was observed in grain biomass for ash alone as compared to the NPKS alone treatment. Yield increases were typically 10–15% for all other ash and NPKS comparisons, regardless of season or location. Dry season production resulted in 10–18% enhancement in grain yield for the Barind, and 13–35% for Madhupur Tracts.

Straw yield differed greatly between season (*P* < 0.001) and treatment (*P* = 0.003), and with season * region interaction (*P* < 0.001). Straw yields in the dry season were ~ 2/3 higher than for the wet season. The two regions behaved similarly with respect to straw yield. Note that straw yield were higher during wet season while grain yields were higher during the dry season.

**Fig. 3:**
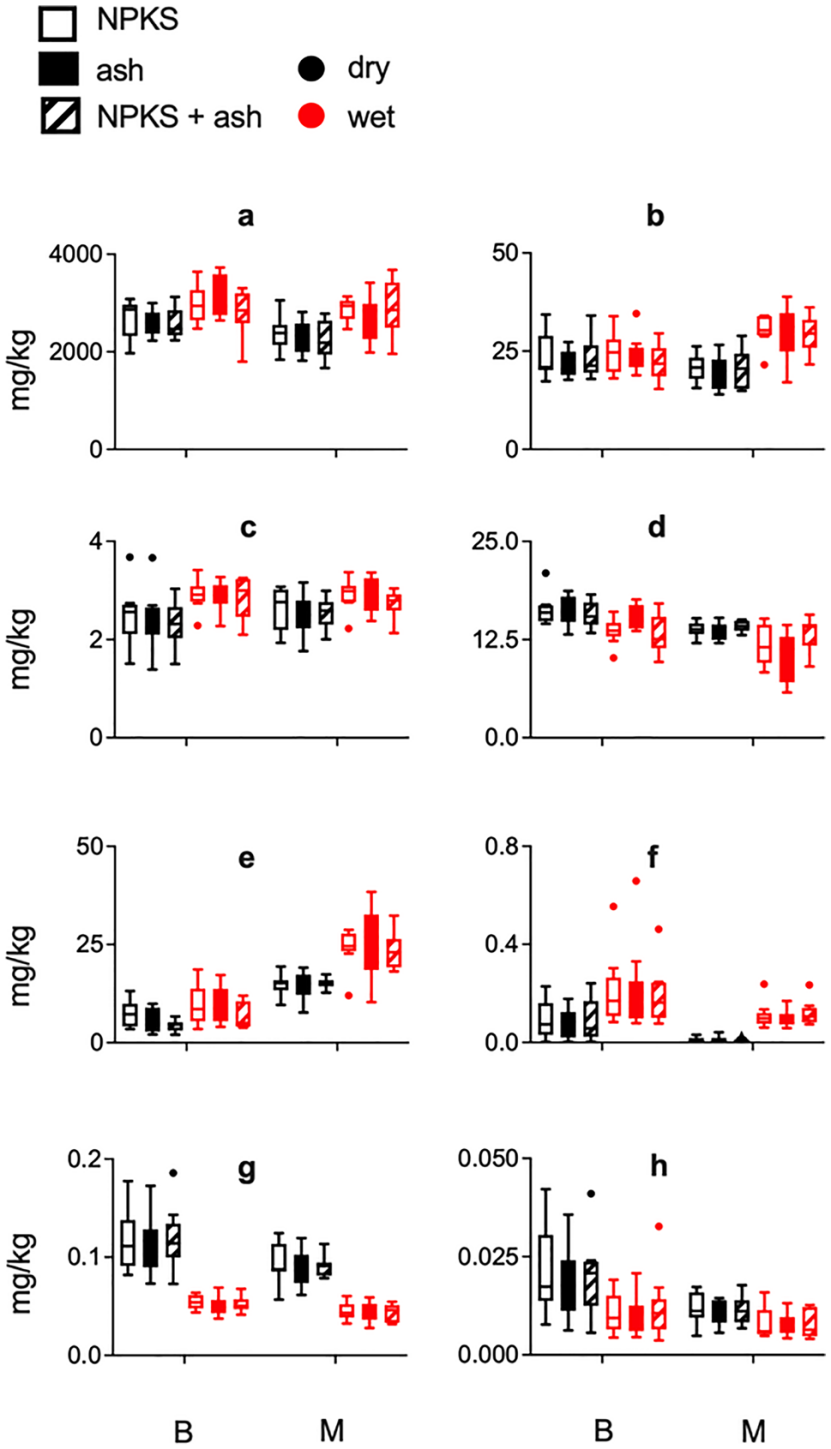
Boxplot for results obtained for rice grain elemental analysis (ICP-MS). Boxplots with whiskers (Tukey) depict results obtained for rice grain elemental analysis (ICP-MS) across all treatment conditions in Madhupur and Barind region in both dry and wet season for elements **a** P, **b** Mn **c** Cu **d** Zn, **e** Rb, **f** Cd, **g** iAs, **h** DMA. *B* Barind region, *M*  Madhupur region, *dry*  dry season, *wet*  wet season, *NPKS*  traditional NPKS treatment, *ash* ash treatment, *NPKS + ash*  traditional NPKS treatment plus ash. For details on significant statistical differences on grain chemical data please see additional file 1 table S5

Elemental concentrations in rice grain differed in response to region for P, Zn, Rb, Cd, iAs, DMA (Fig. 3a, d–h) and season for P, Mn, Cu, Zn, Rb, Cd, iAs, DMA (Fig. 3a–h), but not in response to ash treatment. Only for rice grain Zn, a significant region*treatment effect was observed, with increase due to ash observed in the Barind region during wet season (Fig. 3d). Higher levels of Zn, Cd (*P* < 0.001) and P (*P* < 0.01) were observed for grain collected from the Barind region compared to the Madhupur region. In both regions Cd and P in grain were higher in the wet season (*P* < 0.001), while Zn was higher in the dry season (*P* < 0.001). In the Barind region Cd in rice grain was found to be 0.001–0.24 mg/kg during the dry and 0.08–0.66 mg/kg during the wet season, while in Madhupur it ranged from 0.001–0.04 mg/kg during dry and 0.06–0.24 mg/kg during the wet season. Grain Mn and Rb were particularly high during the wet season in the Madhupur region (significant region*season interaction effect, *P* < 0.001) with grain Rb also overall higher in the Madhupur versus Barind region (*P* < 0.001). In both regions grain iAs and DMA were significantly higher in the dry season (*P* < 0.001), and with these also higher in Barind versus Madhupur region (*P* < 0.001). In the Barind region total As (iAs and DMA) in rice grain ranged from 0.08–0.23 mg/kg during dry and 0.04–0.09 mg/kg during wet season, while in Madhupur it ranged from 0.06–0.14 mg/kg in dry and 0.03–0.07 mg/kg in the wet season. For all statistical results see additional file 1: Table S5.

### Microbial Diversity

All fastq files are publicly available in the European Short Read Archive under accession PRJEB45341. Overall, 108 samples were sequenced and a total of 16,045,926 high-quality 16S rRNA sequences generated, providing a mean of 1,48,573 sequences per sample (additional file 2: Table S6a), with metadata (additional file 2: Table S6b). These generated a count table of 75,086 annotated 16S rRNA ASVs, which were collapsed into 534 genus level ASVs (additional file 3: Table S7). Initial analysis using PERMANOVA analysis identified a significant region (*R*^*2*^ = 0.17*, P* < 0.001), season (*R*^*2*^ = *0.3, P* < *0.01*) but no treatment effect (*R*^*2*^ = *0.01, P* > *0.05*) (additional file 3: Table S8).

**Fig. 4:**
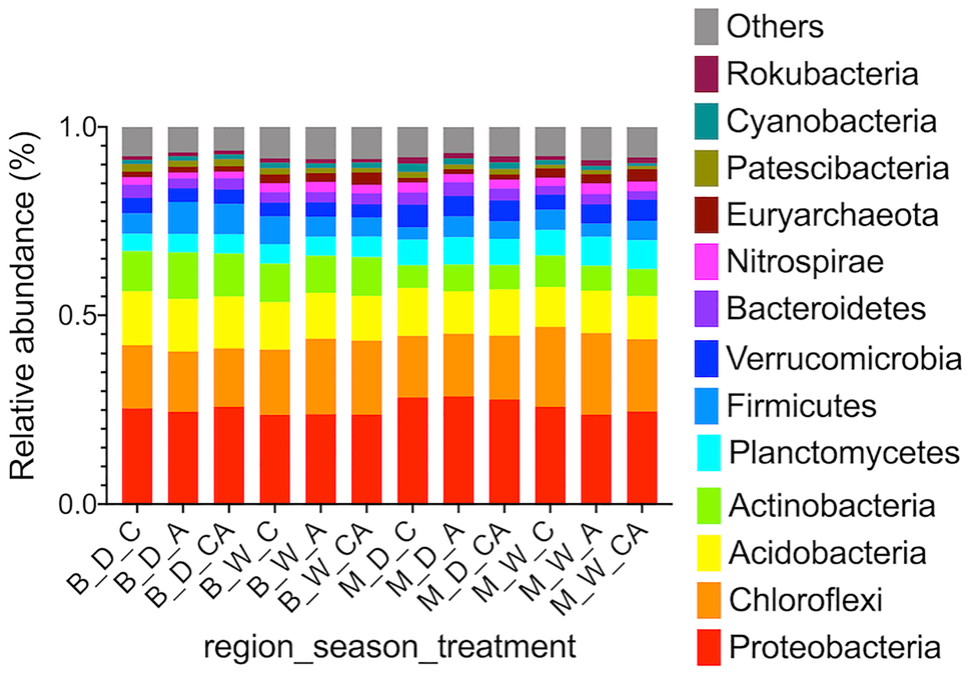
Relative abundance (RA) plot of phylum level ASVs. RA plots showing the dominant 16S rRNA ASVs at phylum level (> 1% RA). Others = the summed RA of less abundant ASVs. *B* Barind region, *M*  Madhupur region, *D*  dry season, *W* wet season, *C*   traditional NPKS treatment, *A*  ash treatment, CA traditional NPKS treatment plus ash

The dominant phyla (> 1% RA) identified were Proteobacteria, Chloroflexi, Acidobacteria, Actinobacteria, Planctomycetes, Verrucomicrobia and Firmicutes (Fig. 4). Actinobacteria, Acidobacteria*,* and Firmicutes were significantly more abundant in the Barind and Planctomycetes and Verrucomicrobia in the Madhupur region. Chloroflexi and Euryarchaeota were significantly more abundant in the wet and Proteobacteria in the dry season (t-test, adjusted P value < 0.01, additional file 3: Table S9).

**Fig. 5:**
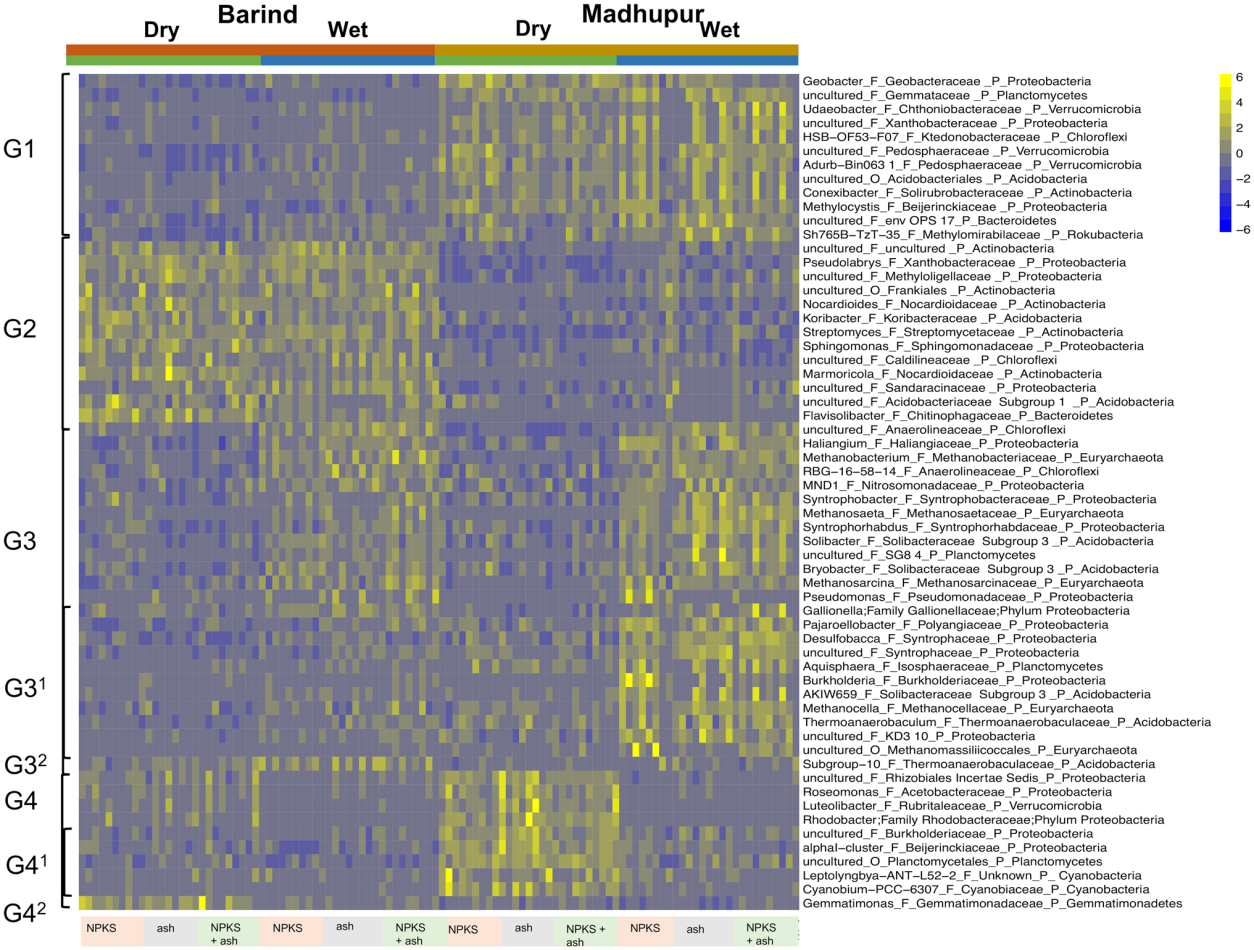
Heatmap showing selected results for significant genus level amplicon sequence varients (ASVs). The heatmap shows selected genus level ASVs with significant effect (DESeq2 analysis) for Madhupur versus Barind (region) and wet versus dry (season). Relative abundance of taxa (centered and scaled) in each row is depicted from blue (lowest) to yellow (highest); G1, G3^1^, G4^1^: higher abundance in Madhupur region; G2, G3^2^, G4^2^: higher abundance in Barind region; G3, G3^1^, G3^2^: higher abundance in wet season; G4, G4^1^, G4^2^: higher abundance in dry season. Sample labels (*NPKS* traditional NPKS treatment, *ash* ash treatment, *NPKS + ash* traditional NPKS treatment) are provided below the heatmap

DESeq2 analysis (Wald test) identified 132 genera level 16S rRNA ASVs with significantly region effect. Of these, 70 showed significantly higher abundance in the Madhupur and 62 in the Barind region. There were 86 genera level 16S rRNA ASVs with significant season effect. Of these 38 showed significantly higher abundance in the dry and 48 in the wet season. Of the 30 most dominant rhizosphere microbial genera, 20 returned a significant DESeq2 result. For the full results table see additional file 3: Table S7, for selected results see Fig. 5.

**Fig. 6:**
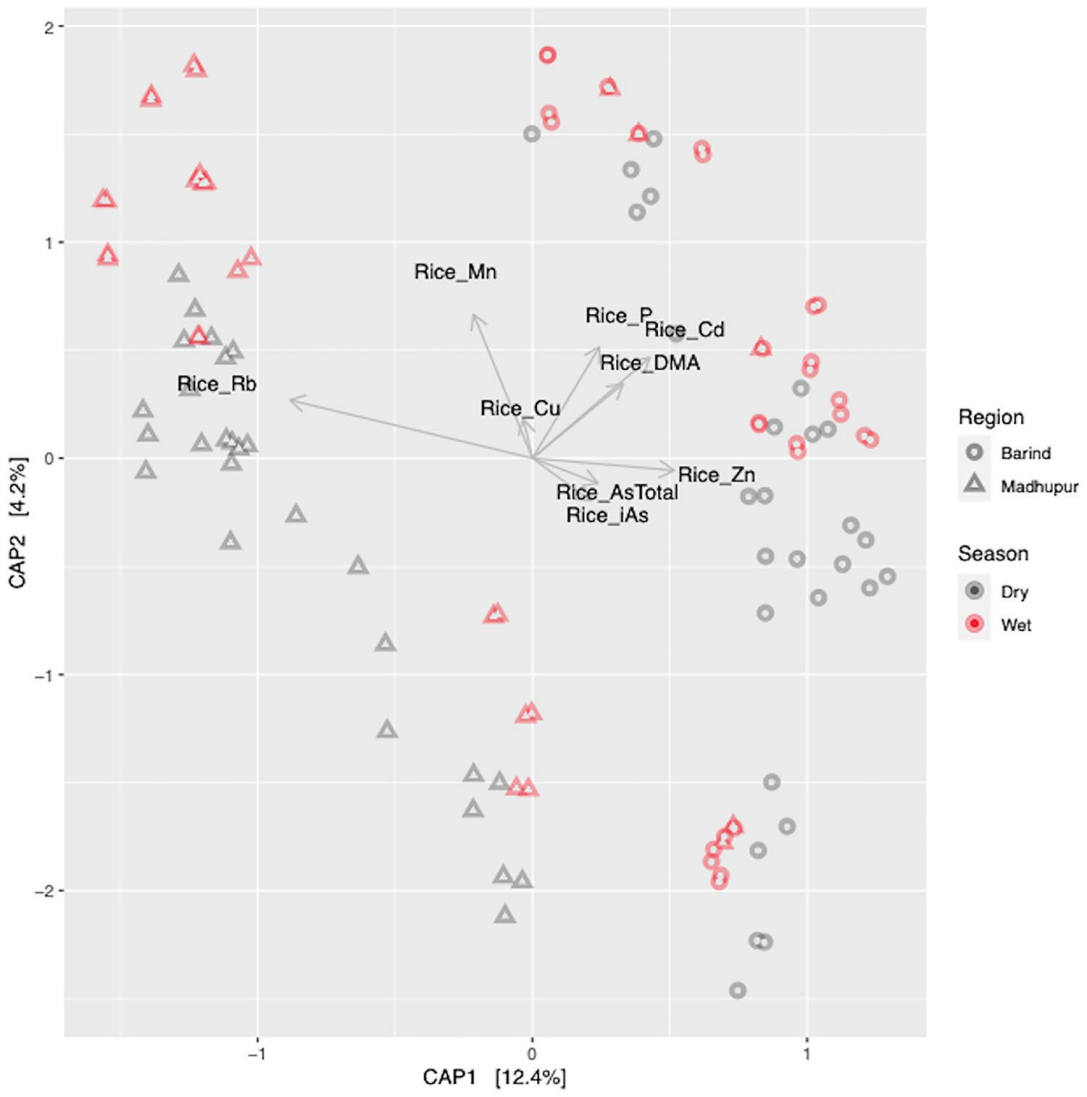
Constrained analysis of principal coordinates (CAP) plot for 16S rRNA and elemental rice grain data. The CAP plot shows the relationship between microbial communities (16S rRNA data) and mineral element concentrations in rice grain. *Barind*  Barind region, *Madhupur *  Madhupur region, *Dry*  dry season, *Wet*  wet season

Grain mineral element variables explained 16.7% of the variation in the microbial community, with the first axis in the Cap plot explaining 12.4% and the second 4.2% (Fig. 6). Grain Rb appears associated with Madhupur, grain P, Zn, Cd, DMA with Barind region.

**Fig. 7:**
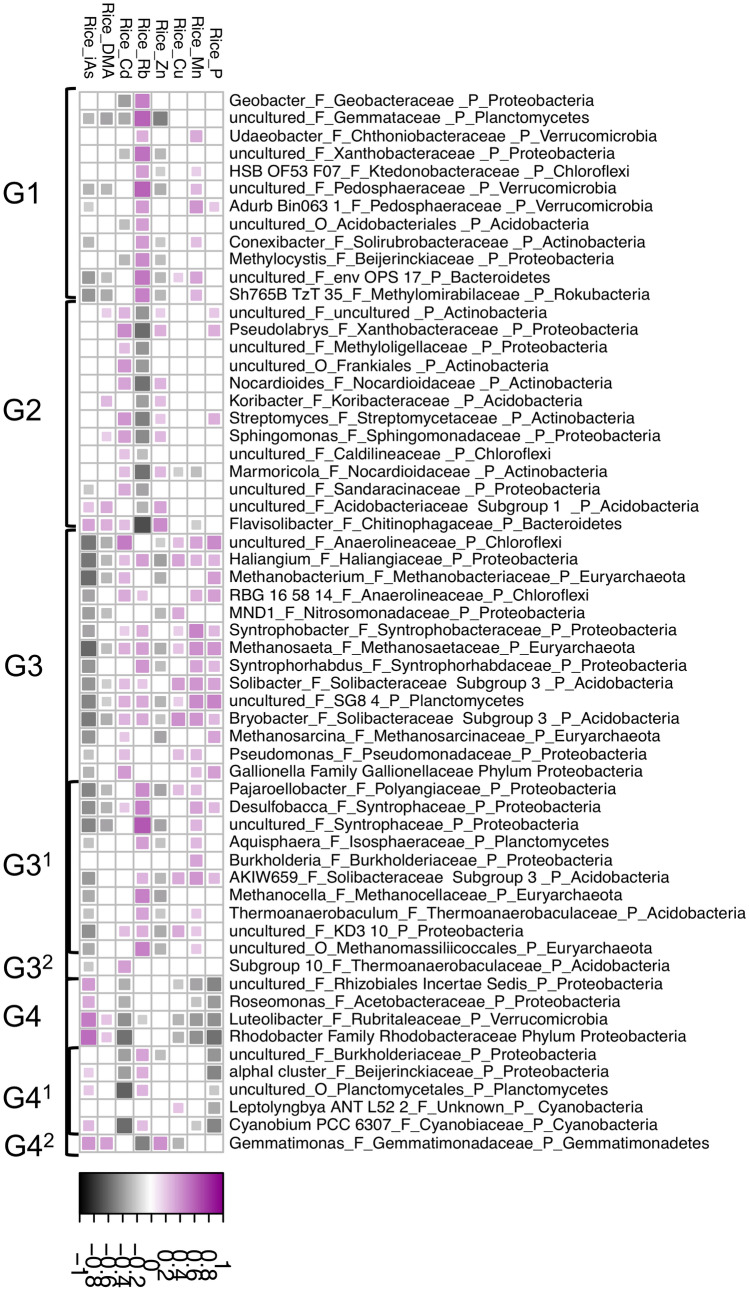
Pairwise Spearman correlations for selected significant genus level ASVs (DESeq2) with rice grain elemental data (ICP-MS). The Spearman correlation plot shows selected significant (*P* < 0.05) correlations between genus level microbial and grain elemental data on a scale from dark pink to dark grey, with dark pink indicating high positive and grey indicating high negative and white no correlation; G1, G3^1^, G4^1^: higher abundance in Madhupur region; G2, G3^2^, G4^2^: higher abundance in Barind region; G3, G3^1^, G3^2^: higher abundance in wet season; G4, G4^1^, G4^2^: higher abundance in dry season

Genera level ASVs elevated in the Barind region tended to be positively correlated with grain Cd, Zn and negatively correlated with grain Rb, while those elevated in the Madhupur region tended to be positively correlated with grain Rb and Mn and negatively correlated with grain Zn. All significant results discussed are shown in Fig. 5 and Fig. 7 and are genera, that have an adjusted FDR < 0.01 for the DESeq2 WALD test (for region or time effect), and with repect to correlation of these genera with grain elemental data, a Spearman Correlation P value < 0.01 with one or more grain elements. Genera level ASVs that showed significant positive correlations with grain elements Cd, Cu, Mn, P, Rb and negative correlations with iAs, DMA, Zn, tended to be more abundant in the wet season, while those that correlated positively with iAs, DMA and negatively with Cd, Mn, P tended to be more abundant in the dry season. For the full results table see additional file 3: Table S7, for selected results see Fig. 7.

## Discussion

In agreement with previous Bangladesh-wide studies it was found that a diverse range of agronomic and arboreal resources are burnt as household fuel (Abedin and Quddus 1990; Jashimuddin et al., 2006; Akther et al., 2010; Braadbaard et al. 2012; Sharma et al. 2015; Thy et al. 2013; Thind et al. 2012; Okon et al. 2005; Van Ryssen et al. 2004) and that the macro-mineral content of biomass ash is a rich source of Ca, K, Mg, P and Si (Bradbaart et al. 2012; Thy et al. 2013; Sharma et al. 2015; Okon et al. 2005; Thind et al. 2012; Van Ryssen et al. 2004). Most Bangladeshi rice cropping systems grow rice twice per year during dry (Boro) and wet (Amon) season and cover an area of > 2,000,000 ha (Islam 2021). Thus, household ash collection could provide the P for one crop per year and a substantial proportion (if not over-supplying in cases) of K requirement, a saving of 50% on P purchase. If ash supplementation of P and K fertilizer is integrated with soil based tests developed for rice cultivation in Bangladesh this could be further optimised (FRG 2012).

In the experiments reported here, not only was yield maintained by reduced fertilizer inputs, it was boosted by 20% through ash amendment and showed the potential to increase grain Zn. Previous trials with rice husk ash (RHA), which is less nutrient rich than household ash, have also shown enhanced yield (Saleque 2002, Thind et al. 2012). Zn deficiency in wetland rice cultures in Bangladesh is widespread and yield increase in response to Zn well documented (Islam 2008). Yield was also enhanced when ash was added to standard NPKS practice, i.e. when nutrients were well oversupplied implicating additional benefits. When farmers were asked why they applied ash, all stated it was for pest control. This may be due to Si, a dominant ash component, as is reported to act as a pest deterrent, microbial and insect (Meharg and Meharg 2015).

Rice grain iAs in the Barind and Madhupur region during the dry season was found to be just above the 0.1 mg/kg European Union recommended threshold for rice in baby food (EU 2015), and this standard has now been set by the USA (FDA 2022). These baby rice standards are not set on rice type (polished or wholemeal) but that an infant (child < 4.5y old) may consume a product that contains rice. Some Barind samples were just above the iAs 0.2 mg/kg recommended threashold for adults in white rice (WHO 2014), but the samples analysed here are wholegrain for which there is still some confusion with standards of iAs 0.4 mg/kg suggested (EU 2015; WHO 2014). In contrast to this, iAs in rice grain collected during the wet season from both regions was well below the 0.1 mg/kg recommended threashold for rice in baby food (EU 2015), at ~ 0.5 mg/kg. For Cd, levels above the internationally recognized standard of 0.2 mg/kg (Shi et al. 2020) were identified in the Barind region during wet season, but not during dry season. Application of ash had no impact on As or Cd in rice grain.

In the current study the dominant bacterial phyla identified across both regions and both seasons, irrespective of treatment, were Proteobacteria, Actinobacteria, Chloroflexi, Firmicutes, Planctomycetales, Acidobacteria, and Verrucomicrobia*,* which is in line with previous reports on microbes in rice rhizosphere soil (Edwards et al. 2019; Hernández et al. 2015). Paddy soil microbial communities were shown to vary most strongly in response to region, followed by season, but showed no effect in response to household biomass ash treatment. As this study was limited to application of ash over 2 seasons, impact of longer term application of household ash on the abundance of rhizosphere soil microbes cannot as yet be ruled out.

With respect to regional differences, rice grains in the Barind region were elevated in Cd, Zn, P when compared to Madhupur region. This coincided with higher soil pH (around 5.5) and higher abundance of aerobic *Pseudolabrys* (Kämpfer et al. 2006) and microbes previously reported to occur in paddy soils with elevated Cd such as *Marmoricola* and *Nocardioiles* (Song et al. 2020), *Gemmatimonas* (An et al. 2021), *Flavisolibacter* (Liu et al. 2020) and *Sphingomonas* (Nilgiriwara et al. 2008). *Flavisolibacter* have previously been shown to catalyze hydrogen peroxide to protect itself and host plants from high Cd contamination (Liu et al. 2020), and *Sphingomonas* to express high levels of alkaline phosphatase, which bioprecipitate Cd present in soils and resist its phytotoxicity (Nilgiriwara et al. 2008). Rice grain in the Madhupur region showed elevated levels of Rb. This coincided with lower soil pH (around 5) and higher abundance of aerobic *Conexibacter*, anaerobic *Geobacter* and methanotroph *Methylocystis* amongst others*. Geobacter* are known to perform Fe(III) reduction and are reported as primary agents that can couple the oxidation of organic compounds to the reduction of insoluable Fe (III) and Mn (IV) oxides (Aklujkar et al. 2013; Lovley et al. 2011)*.* The methanotroph *Methylocystis* has previously been reported as the predominant methanotroph in paddy soils with pH of around 5 (Shiau et al. 2018). With respect to seasonal differences, there was an increase in rice grain Cd, Cu, Mn, P, Rb observed during wet and rice grain iAs, DMA, Zn during dry season. Further to that, *Gallionella, Haliangium*, *Pseudomonas* and genera within the family *Anaerolineaceae* (phylum Chloroflexi) and methanogenic archaea were particularly abundant during wet season and showed positive correlation with Cd, Mn, P and negative correlation with iAs, DMA concentration in rice grain. Higher concentrations of Cd in rice grain in the wet season and iAs, DMA in the dry season have previously been reported in several other studies (Islam et al. 2018; Jahiruddin et al. 2017; Xu et al. 2008). *Anaerolineaceae* are anaerobes with a role in degrading organic matter (Sinkko et al. 2013). They may release Cd, Mn, P from organic material and thereby increase uptake into the rice grain. Higher abundance of Fe-oxidizing bacteria (FeOB) like *Gallionella* and *Pseudomonas* in wet season may contribute to the immobilization of As via formation of solid Fe (hydro)oxides minerals (Emerson et al. 2013; Razzak et al. 2021). This immobilization may contribute to lower levels of As in wet versus dry season. *Rhodobacter*, which are arsenic methylating bacteria, showed positive correlation with grain iAs, DMA and were more abundant in the dry season. Methanogenic archaea, known to be involved in As demethylation (Chen et al. 2019) showed negative correlation with grain iAs, DMA and higher abundance during the wet season. Hence, these organisms, and others not assessed by the analysis approaches used here, may contribute to the observed higher levels of grain iAs, DMA in the dry season.

This study demonstrated that rice yield increase in response to ash treatment, whilst maintaining rice grain quality and with no negative impact on soil microbes, providing a route to more sustainable rice cultivation and implicates a potential role of microbes in the observed regional and seasonal differences in rice grain elements. Results should be further investigated in longer-term studies.

### Supplementary Information

Below is the link to the electronic supplementary material.

Supplementary file1 (DOCX 2887 KB)

Supplementary file2 (XLSX 29 KB)

Supplementary file3 (XLSX 1022 KB)

## Data Availability

Data generated or analysed during this study are included in this article and its supplementary information files. The raw sequencing data (fastq files) have been submitted to the European Short Read Archive and can be accessed under accession PRJEB45341.
