# Supplementary material for: Recycled Household Ash in Rice Paddies of Bangladesh for Sustainable Production of Rice Without Altering Grain Arsenic and Cadmium
Source: Expo Health. 2023 Feb 9;16(1):87–99. doi: 10.1007/s12403-023-00539-y (PMC10830805; doi:10.1007/s12403-023-00539-y)
Supplement: Supplementary file 1 — Supplementary file1 (DOCX 2887 KB) [file 12403_2023_539_MOESM1_ESM.docx]

**Abbreviations**

ASVs: Amplicon sequencing variants; PERMANOVA: Permutational multivariate analysis of variation; QIIME: Quantitative Insights Into Microbial Ecology; FeOB: Fe-oxidizing bacteria; DMA: Dimethylarsinic acid; RHA: Rice husk ash; ICP-MS: Inductively coupled plasma mass spectrometry; XRF: X-ray fluorescence

**Table S1** Elemental recovery of CRMs by XRF

|  |  |  | | **certified value** | **average** | **stand. dev.** | **recovery** |  |
| --- | --- | --- | --- | --- | --- | --- | --- | --- |
|  |  |  | | (mg/kg) | (mg/kg) | (mg/kg) | (%) |  |
|  |  | |  |  |  |  |  |  |
| **CRM** | **NCS 73007** | | (n=27) |  |  |  |  |  |
|  |  | |  |  |  |  |  |  |
|  |  | **Al** | | 94471.2 | 121296.3 | 724.0 | 128.4 |  |
|  |  | **As** | | 18.0 | 20.0 | 1.4 | 111.0 |  |
|  |  | **Ba** | | 411.0 | 371.9 | 20.4 | 90.5 |  |
|  |  | **Ca** | | 2858.8 | 2971.5 | 95.2 | 103.9 |  |
|  |  | **Cu** | | 32.0 | 39.4 | 3.4 | 123.0 |  |
|  |  | **Fe** | | 38048.7 | 37966.7 | 188.1 | 99.8 |  |
|  |  | **K** | | 20753.7 | 22511.1 | 544.4 | 108.5 |  |
|  |  | **Mg** | | 5065.5 | 7912.6 | 439.3 | 156.2 |  |
|  |  | **Mn** | | 441.0 | 486.2 | 12.3 | 110.3 |  |
|  |  | **Ni** | | 27.4 | 15.0 | 4.9 | 54.6 |  |
|  |  | **P** | | 972.0 | 1088.9 | 19.1 | 112.0 |  |
|  |  | **Rb** | | 173.0 | 187.4 | 1.5 | 108.3 |  |
|  |  | **S** | | 261.0 | 408.5 | 21.0 | 156.5 |  |
|  |  | **Sr** | | 68.0 | 79.2 | 1.4 | 116.5 |  |
|  |  | **Si** | | 298223.5 | 288518.5 | 1868.2 | 96.7 |  |
|  |  | **Ti** | | 5780.0 | 5978.5 | 97.9 | 103.4 |  |
|  |  | **Zn** | | 100.0 | 110.6 | 3.5 | 110.6 |  |
|  |  |  | |  |  |  |  |  |
|  |  |  | |  |  |  |  |  |
| **CRM** | **ISE921** | (n=34) | |  |  |  |  |  |
|  |  |  | |  |  |  |  |  |
|  |  | **Al** | | 56800.0 | 72411.8 | 1981.9 | 127.5 |  |
|  |  | **As** | | 29.9 | 33.8 | 1.6 | 113.0 |  |
|  |  | **Ba** | | 563.0 | 556.2 | 24.1 | 98.8 |  |
|  |  | **Ca** | | 43000.0 | 44767.6 | 915.8 | 104.1 |  |
|  |  | **Cu** | | 93.8 | 122.6 | 7.4 | 130.7 |  |
|  |  | **Fe** | | 31900.0 | 32867.6 | 336.4 | 103.0 |  |
|  |  | **K** | | 19100.0 | 18858.8 | 528.6 | 98.7 |  |
|  |  | **Mg** | | 11100.0 | 13497.1 | 815.6 | 121.6 |  |
|  |  | **Mn** | | 1190.0 | 1289.1 | 26.4 | 108.3 |  |
|  |  | **Ni** | | 42.4 | 51.8 | 8.5 | 122.1 |  |
|  |  | **P** | | 1400.0 | 1600.0 | 82.6 | 114.3 |  |
|  |  | **Rb** | | 103.0 | 116.4 | 1.5 | 113.0 |  |
|  |  | **S** | | 702.0 | 1085.4 | 53.2 | 154.6 |  |
|  |  | **Sr** | | 271000.0 | 257705.9 | 7918.3 | 95.1 |  |
|  |  | **Si** | | 165.0 | 179.6 | 2.5 | 108.9 |  |
|  |  | **Ti** | | 3700.0 | 3626.8 | 111.5 | 98.0 |  |
|  |  | **Zn** | | 522.0 | 582.1 | 8.6 | 111.5 |  |
| **CRM** | **NIST 1568b** | (n=13) | |  |  |  |  |  |
|  |  |  | |  |  |  |  |  |
|  |  | **iAs** | | 0.092 | 0.087 | 0.010 | 94.5 |  |
|  |  | **DMA** | | 0.180 | 0.171 | 0.017 | 94.8 |  |
|  |  | **P** | | 1530 | 1625 | 259 | 106.2 |  |
|  |  | **Mn** | | 19.2 | 21.8 | 2.24 | 113.3 |  |
|  |  | **Cu** | | 2.35 | 2.38 | 0.27 | 101.5 |  |
|  |  | **Zn** | | 19.2 | 14.5 | 0.80 | 76.4 |  |
|  |  | **Rb** | | 6.20 | 5.84 | 0.35 | 94.2 |  |

**Table S2** Fuels used in domestic cooking per household per day, along with ash produced, ash use per household and size of plot cultivated. Kruskal-Wallace analysis significance is report for between tract comparisons are given at the: 5 (*), 1 (**) and 0.1 (***) % level, respectively

| **Category** | **Region** | **min.** | **25%** | **median** | **75%** | **max.** | **signif. (P)** |
| --- | --- | --- | --- | --- | --- | --- | --- |
| income (Tk) | Barind | 19000 | 100000 | 200000 | 320000 | 1700000 | *** |
|  | Madhupur | 0 | 74000 | 120000 | 220000 | 1200000 |  |
| farm size (ha) | Barind | 0.00 | 0.27 | 0.54 | 1.33 | 6.92 | * |
|  | Madhupur | 0.00 | 0.26 | 0.40 | 0.81 | 4.84 |  |
| family size (n) | Barind | 2 | 4 | 4 | 5 | 14 |  |
|  | Madhupur | 2 | 4 | 5 | 5 | 11 |  |
| cow dung (kg/d) | Barind | 0.00 | 0.00 | 2.00 | 5.00 | 40.00 | *** |
|  | Madhupur | 0.00 | 0.00 | 0.00 | 0.00 | 13.00 |  |
| straw (kg/d) | Barind | 0.00 | 0.00 | 1.00 | 2.25 | 20.00 | *** |
|  | Madhupur | 0.00 | 0.00 | 0.00 | 0.00 | 15.00 |  |
| branches (kg/d) | Barind | 0.00 | 0.00 | 1.00 | 3.00 | 20.00 | *** |
|  | Madhupur | 0.00 | 3.00 | 4.00 | 5.00 | 20.00 |  |
| leaves (kg/d) | Barind | 0.00 | 0.00 | 0.20 | 2.00 | 15.00 | *** |
|  | Madhupur | 0.00 | 1.00 | 2.00 | 2.00 | 40.00 |  |
| husk/bran (kg/d) | Barind | 0.00 | 0.00 | 0.00 | 0.50 | 5.00 | *** |
|  | Madhupur | 0.00 | 0.00 | 0.00 | 0.00 | 0.00 |  |
| crop residue (kg/d) | Barind | 0.00 | 0.00 | 0.00 | 0.00 | 5.00 |  |
|  | Madhupur | 0.00 | 0.00 | 0.00 | 0.00 | 0.00 |  |
| firewood (kg/d) | Barind | 0.00 | 0.00 | 0.00 | 0.00 | 12.00 | *** |
|  | Madhupur | 0.00 | 0.00 | 0.00 | 2.00 | 30.00 |  |
| jute (kg/d) | Barind | 0.00 | 0.00 | 0.00 | 0.00 | 20.00 |  |
|  | Madhupur | 0.00 | 0.00 | 0.00 | 0.00 | 0.50 |  |
| bamboo (kg/d) | Barind | 0.00 | 0.00 | 0.00 | 1.00 | 10.00 | *** |
|  | Madhupur | 0.00 | 0.00 | 0.00 | 0.00 | 3.00 |  |
| sum fuel (kg/d) | Barind | 0.00 | 6.00 | 9.00 | 14.00 | 85.00 | *** |
|  | Madhupur | 0.00 | 6.00 | 7.00 | 9.50 | 48.00 |  |
| ash produced (t/y) | Barind | 0.04 | 0.31 | 0.58 | 0.95 | 4.60 | *** |
|  | Madhupur | 0.04 | 0.22 | 0.26 | 0.30 | 1.79 |  |
| ash use (t/y) | Barind | 0.00 | 0.00 | 0.20 | 1.20 | 30.00 | *** |
|  | Madhupur | 0.00 | 0.00 | 0.00 | 0.12 | 5.04 |  |
| ash use (t/ha) | Barind | 0.00 | 0.00 | 0.30 | 3.00 | 32.23 | *** |
|  | Madhupur | 0.00 | 0.00 | 0.00 | 0.29 | 19.54 |  |
| ash available (t/ha) | Barind | 0.00 | 0.34 | 0.83 | 2.13 | 22.38 | ** |
|  | Madhupur | 0.01 | 0.27 | 0.56 | 1.07 | 10.14 |  |

**Table S3** Domestic ash chemistry for averaged by region. All units, excluding pH are in mg/kg. For each element the mean concentration is given and underneath this concentration is the standard error of the mean. Probabilities for a two-way ANOVA are given at the: 5 (*), 1 (**) and 0.1 (***) % level, respectively

|  |  |  |  |  |  |  |  |  | **probability** |  |
| --- | --- | --- | --- | --- | --- | --- | --- | --- | --- | --- |
| **region** |  | **Barind** | |  | **Madhupur** | |  | **region** | **season** | **region x** |
| **season** |  | **dry** | **wet** |  | **dry** | **wet** |  |  |  | **season** |
| Al |  | 16682 | 16807 |  | 19746 | 18339 |  | * |  |  |
|  |  | 848 | 807 |  | 1030 | 696 |  |  |  |  |
| As |  | 4.18 | 4.11 |  | 4.95 | 5.15 |  | *** |  |  |
|  |  | 0.21 | 0.16 |  | 0.32 | 0.12 |  |  |  |  |
| Ba |  | 402 | 426 |  | 642 | 659 |  | *** |  |  |
|  |  | 11 | 12 |  | 19 | 25 |  |  |  |  |
| Ca |  | 42210 | 39435 |  | 142005 | 148813 |  | *** |  |  |
|  |  | 1777 | 1590 |  | 6176 | 4383 |  |  |  |  |
| Cu |  | 47.1 | 46.2 |  | 124.1 | 102.6 |  | *** |  |  |
|  |  | 1.4 | 1.4 |  | 17.6 | 4.4 |  |  |  |  |
| Fe |  | 7244 | 7676 |  | 10767 | 9997 |  | *** |  |  |
|  |  | 311 | 293 |  | 614 | 380 |  |  |  |  |
| K |  | 58838 | 61935 |  | 45235 | 50363 |  | *** |  |  |
|  |  | 2256 | 2172 |  | 2287 | 1384 |  |  |  |  |
| Mg |  | 21297 | 19424 |  | 41060 | 37944 |  | *** | * |  |
|  |  | 664 | 516 |  | 1694 | 1578 |  |  |  |  |
| Mn |  | 3268 | 2847 |  | 2603 | 2520 |  | *** |  |  |
|  |  | 126 | 60 |  | 179 | 134 |  |  |  |  |
| Ni |  | 15.7 | 21.6 |  | 35.9 | 42.9 |  | *** | *** |  |
|  |  | 0.2 | 0.5 |  | 1.3 | 1.5 |  |  |  |  |
| P |  | 11916 | 9805 |  | 11099 | 12482 |  |  |  | *** |
|  |  | 564 | 288 |  | 703 | 377 |  |  |  |  |
| Rb |  | 159 | 143 |  | 213 | 233 |  | *** |  |  |
|  |  | 4 | 5 |  | 13 | 11 |  |  |  |  |
| S |  | 3737 | 3734 |  | 6912 | 7439 |  | *** |  |  |
|  |  | 85 | 147 |  | 261 | 358 |  |  |  |  |
| Si |  | 320081 | 306339 |  | 179180 | 170912 |  | *** | * |  |
|  |  | 3443 | 3721 |  | 6762 | 4996 |  |  |  |  |
| Sr |  | 151 | 147 |  | 466 | 523 |  | *** |  |  |
|  |  | 7 | 7 |  | 30 | 33 |  |  |  |  |
| Ti |  | 1157 | 1357 |  | 1365 | 1397 |  |  |  |  |
|  |  | 73 | 57 |  | 189 | 68 |  |  |  |  |
| Zn |  | 414 | 413 |  | 583 | 496 |  | ** |  |  |
|  |  | 18 | 23 |  | 82 | 17 |  |  |  |  |

**Fig.S1** PCA analysis of individual household fuel usage per household for Barind and Madhupur. The small black filled squares are the factor analysis for the substrates burnt

**
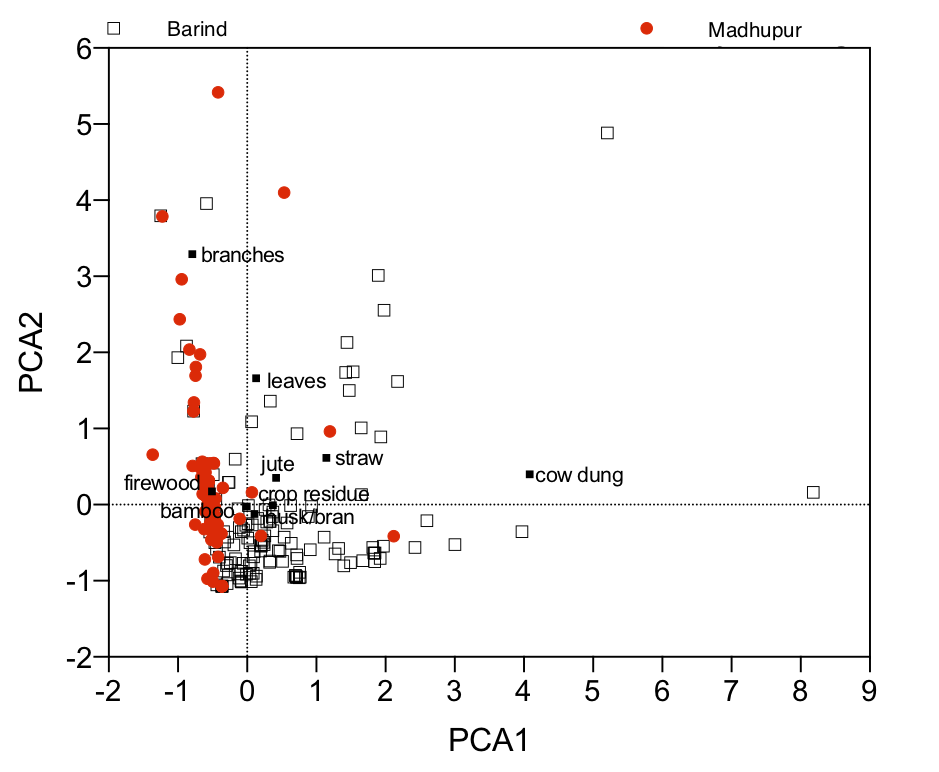
**

**Table S4** Two-factor ANOVA (treatment, region) and post hoc for soil elemental data (XRF)

|  |  | **Post Hoc Tukey Test** | | | | | | **Anova*** | | |
| --- | --- | --- | --- | --- | --- | --- | --- | --- | --- | --- |
|  |  | **Barind**  mg/kg (except for pH) | | | **Madhupur**  mg/kg (except for pH) | | | **Main effect** | | **Interaction effect** |
| **Element** | **Value** | **C** | **A** | **CA** | **C** | **A** | **CA** | **R** | **T** | **R*T** |
| **Al** | means | 59304 | 60356 | 58381 | 70741 | 70226 | 70319 | *** | n.a. | n.a. |
|  | groups | a | a | a | a | a | a |  |  |  |
| **iAs** | means | 3.46 | 4.07 | 3.36 | 6.67 | 6.76 | 6.08 | *** | n.a. | n.a. |
|  | groups | c | bc | c | a | a | ab |  |  |  |
| **Ba** | means | 325.52 | 325.89 | 321.70 | 328.96 | 325.52 | 326.07 | n.a. | n.a. | n.a. |
|  | groups | a | a | a | a | a | a |  |  |  |
| **Ca** | means | 2288 | 2360 | 2283 | 3731 | 3711 | 3788 | * | n.a. | n.a. |
|  | groups | a | a | a | a | a | a |  |  |  |
| **Cl**^[np]^ | medians | 56.13 | 66.67 | 53.63 | 32.17 | 24.20 | 31.30 | *** | n.a. | n.a. |
|  | groups | a | abc | ab | bcd | d | cd |  |  |  |
| **Cu** | means | 24.08 | 24.30 | 24.01 | 29.53 | 27.91 | 28.90 | *** | n.a. | n.a. |
|  | groups | a | a | a | a | a | a |  |  |  |
| **Fe** | means | 15544 | 16052 | 15274 | 25122 | 25130 | 24507 | *** | n.a. | n.a. |
|  | groups | b | b | b | a | a | a |  |  |  |
| **K** | means | 17167 | 17133 | 17037 | 14703 | 14683 | 14678 | *** | n.a. | n.a. |
|  | groups | a | a | a | a | a | a |  |  |  |
| **Mg** | means | 5795 | 5862 | 5604 | 6637 | 6433 | 6379 | n.a. | n.a. | n.a. |
|  | groups | a | a | a | a | a | a |  |  |  |
| **Mn** | means | 320.07 | 326.41 | 306.67 | 393.33 | 407.74 | 345.04 | n.a. | n.a. | n.a. |
|  | groups | a | a | a | a | a | a |  |  |  |
| **Ni**^[np]^ | medians | 15.53 | 15.40 | 15.80 | 20.97 | 19.27 | 20.97 | *** | n.a. | n.a. |
|  | groups | b | B | b | a | a | a |  |  |  |
| **P**^[np]^ | medians | 771.00 | 756.67 | 684.00 | 376.33 | 343.67 | 387.00 | *** | n.a. | n.a. |
|  | groups | a | A | a | b | b | b |  |  |  |
| **Rb** | means | 99.14 | 101.26 | 98.71 | 98.33 | 96.67 | 98.97 | n.a. | n.a. | n.a. |
|  | groups | a | a | a | a | a | a |  |  |  |
| **S** | means | 200.81 | 194.30 | 197.48 | 171.14 | 177.23 | 179.21 | n.a. | n.a. | n.a. |
|  | groups | a | a | a | a | a | a |  |  |  |
| **Si**^[np]^ | medians | 334333 | 329667 | 332667 | 302333 | 293333 | 297667 | *** | n.a. | n.a. |
|  | groups | a | ab | a | ab | b | b |  |  |  |
| **Sr** | means | 54.71 | 54.99 | 55.06 | 73.41 | 72.70 | 73.30 | * | n.a. | n.a. |
|  | groups | a | a | a | a | a | a |  |  |  |
| **Zn** | means | 66.14 | 63.73 | 72.18 | 63.99 | 60.34 | 64.41 | n.a. | n.a. | n.a. |
|  | groups | a | a | a | a | a | a |  |  |  |
| **pH** | means | 5.65 | 5.57 | 5.67 | 5.18 | 5.26 | 5.25 | ** | n.a. | n.a. |
|  | groups | a | a | a | a | a | a |  |  |  |

Two-factor ANOVA (R=region, T=treatment, n=9) and post hoc (Tukey) results obtained for soil elemental data (XRF). Treatments: C=NPKS, A=ash, CA=NPKS+ ash (soil samples collected at end of experiment). For non-parametric data^[np]^, ANOVA and Tukey test were performed on ranked data. Significance codes: very highly significant p<0.001 ‘***’; highly significant p<0.01‘**’; significant p<0.05 ‘*’; not significant p>0.05 ‘ n.s.’. Means/medians*: calculated from 9 replicate samples. Groups: Tukey results for multiple pairwise comparisons, where conditions that do not share a letter are considered significant at P<0.05 after correction for multiple testing (Tukey)

**Table S5** Three-factor ANOVA (treatment, season, region) and post hoc results for rice grain elemental data (ICP-MS).

|  |  | **Post Hoc Tukey Test** | | | | | | | | | | | | **Anova** | | | | | | |
| --- | --- | --- | --- | --- | --- | --- | --- | --- | --- | --- | --- | --- | --- | --- | --- | --- | --- | --- | --- | --- |
| **Rice** |  | **Barind** (mg/kg) | | | | | | **Madhupur** (mg/kg) | | | | | |  |  |  |  |  |  |  |
|  |  | **Dry** | | | **Wet** | | | **Dry** | | | **Wet** | | | **Main effect** | | | **Interaction effect** | | | |
| **Element** | **Value** | **C** | **A** | **CA** | **C** | **A** | **CA** | **C** | **A** | **CA** | **C** | **A** | **CA** | **R** | **S** | **T** | **R*S** | **R*T** | **S*T** | **R*S*T** |
| **Zn** | mean | 16.27 | 16.25 | 15.72 | 13.60 | 15.32 | 13.16 | 13.80 | 13.65 | 14.23 | 11.81 | 10.46 | 13.27 | *** | *** | n.s | n.s | ** | n.s | n.s |
|  | groups | a | a | ab | abc | ab | bcd | abc | abc | abc | cd | d | abcd |  |  |  |  |  |  |  |
| t-test: | A vs. C | n.s | |  | * | |  | n.s | |  | n.s | |  |  |  |  |  |  |  |  |
| **Rb** | mean | 7.38 | 5.61 | 4.29 | 9.79 | 9.33 | 6.74 | 14.74 | 14.59 | 15.09 | 24.05 | 25.49 | 23.50 | *** | *** | n.s | *** | n.s | n.s | n.s |
|  | groups | c | c | c | bc | bc | c | b | b | b | a | a | a |  |  |  |  |  |  |  |
| **P** | mean | 2645 | 2561 | 2570 | 2988 | 3130 | 2825 | 2372 | 2295 | 2239 | 2861 | 2677 | 2895 | ** | *** | n.s | n.s | n.s | n.s | n.s |
|  | groups | abc | abc | abc | a | a | abc | bc | bc | c | ab | abc | ab |  |  |  |  |  |  |  |
| **Mn** | mean | 23.63 | 21.68 | 23.20 | 24.26 | 24.09 | 22.23 | 20.65 | 19.58 | 20.51 | 30.32 | 29.63 | 29.22 | n.s | *** | n.s | *** | n.s | n.s | n.s |
|  | groups | abcd | cd | abcd | abcd | abcd | bcd | d | d | d | a | ab | abc |  |  |  |  |  |  |  |
| **Cu** | mean | 2.49 | 2.40 | 2.32 | 2.91 | 2.87 | 2.83 | 2.62 | 2.52 | 2.53 | 2.91 | 2.94 | 2.72 | n.s | *** | n.s | n.s | n.s | n.s | n.s |
|  | groups | a | a | a | a | a | a | a | a | a | a | a | a |  |  |  |  |  |  |  |
| **Cd**^[np]^ | median | 0.07 | 0.08 | 0.06 | 0.17 | 0.14 | 0.16 | 0.01 | 0.01 | 0.01 | 0.10 | 0.08 | 0.11 | *** | *** | n.s | n.s | n.s | n.s | n.s |
|  | groups | ab | abc | abc | a | a | a | ab | abc | abc | bc | c | c |  |  |  |  |  |  |  |
| **iAs**^[np]^ | median | 0.11 | 0.12 | 0.11 | 0.05 | 0.05 | 0.05 | 0.09 | 0.09 | 0.09 | 0.04 | 0.04 | 0.05 | *** | *** | n.s | n.s | n.s | n.s | n.s |
|  | groups | a | a | a | b | b | b | a | a | a | b | b | b |  |  |  |  |  |  |  |
| **DMA**^[np]^ | median | 0.02 | 0.02 | 0.02 | 0.01 | 0.01 | 0.01 | 0.01 | 0.01 | 0.01 | 0.01 | 0.01 | 0.01 | *** | *** | n.s | n.s | n.s | n.s | n.s |
|  | groups | a | a | a | ab | ab | ab | ab | ab | ab | b | b | b |  |  |  |  |  |  |  |

Three-factor ANOVA (R=region, S=season, T=treatment, n=9) and post hoc (Tukey) results obtained for rice grain elemental data (ICP-MS). For Zn a post hoc t-test for A versus C within each Region-Season was conducted to further explore the significant Region*Treatment effect. Season: dry= boro, irrigated, wet = aman, rainfed. Treatments: C=NPKS, A=ash, CA=NPKS+ ash. For non-parametric data^[np]^, ANOVA and Tukey test were performed on ranked data. For Zn, the result (P value) for the t-test for C versus A is shown in row t-test. Significance codes: very highly significant p<0.001 ‘***’; highly significant p<0.01‘**’; significant p<0.05 ‘*’; not significant p>0.05 ‘ n.s.’. Means/medians*: calculated from 9 replicate samples. Groups: Tukey results for multiple pairwise comparisons, where conditions that do not share a letter are considered significant at P<0.05 after correction for multiple testing (Tukey)
